# *Methanomethylophilus alvi* gen. nov., sp. nov., a Novel Hydrogenotrophic Methyl-Reducing Methanogenic Archaea of the Order *Methanomassiliicoccales* Isolated from the Human Gut and Proposal of the Novel Family *Methanomethylophilaceae* fam. nov.

**DOI:** 10.3390/microorganisms11112794

**Published:** 2023-11-17

**Authors:** Guillaume Borrel, Khaled Fadhlaoui, Wajdi Ben Hania, Nadia Gaci, Gérard Pehau-Arnaudet, Prem Prashant Chaudhary, Pascal Vandekerckove, Nathalie Ballet, Monique Alric, Paul William O’Toole, Marie-Laure Fardeau, Bernard Ollivier, Jean-François Brugère

**Affiliations:** 1 Institut Pasteur, Université Paris Cité, Evolutionary Biology of the Microbial Cell, 75015 Paris, France; 2 Aix Marseille Univ., Université de Toulon, CNRS, IRD, MIO, 13288 Marseille, France; khaled.fadhlaoui@uca.fr (K.F.); bernard.ollivier@mio.osupytheas.fr (B.O.); 3 Université Clermont Auvergne, INRA, MEDIS, 63000 Clermont-Ferrand, France; 4 Université Clermont Auvergne, CNRS, UMR 6023 CNRS-UCA, Laboratoire Microorganismes: Génome et Environnement LMGE, 63000 Clermont-Ferrand, France; 5 Université d’Auvergne, EA CIDAM, 63000 Clermont-Ferrand, Francej-francois.brugere@uca.fr (J.-F.B.); 6 Institut Pasteur, Université Paris Cité, Ultrastructural Bioimaging, 75015 Paris, France; 7 Epithelial Therapeutics Unit, National Institute of Allergy and Infectious Diseases, National Institutes of Health, Bethesda, MD 20892, USA; 8 Lesaffre International, Lesaffre Group, 59700 Marcq-en-Barœul, France; 9 APC Microbiome Ireland, University College Cork, T12 K8AF Cork, Ireland

**Keywords:** archaea, methanogens, human gut microbiota, *Methanomassiliicoccales*

## Abstract

The methanogenic strain Mx-05^T^ was isolated from the human fecal microbiome. A phylogenetic analysis based on the 16S rRNA gene and protein marker genes indicated that the strain is affiliated with the order *Methanomassiliicoccales*. It shares 86.9% 16S rRNA gene sequence identity with *Methanomassiliicoccus luminyensis*, the only member of this order previously isolated. The cells of Mx-05^T^ were non-motile cocci, with a diameter range of 0.4–0.7 μm. They grew anaerobically and reduced methanol, monomethylamine, dimethylamine, and trimethylamine into methane, using H_2_ as an electron donor. H_2_/CO_2_, formate, ethanol, and acetate were not used as energy sources. The growth of Mx-05^T^ required an unknown medium factor(s) provided by *Eggerthella lenta* and present in rumen fluid. Mx-05^T^ grew between 30 °C and 40 °C (optimum 37 °C), over a pH range of 6.9–8.3 (optimum pH 7.5), and between 0.02 and 0.34 mol.L^−1^ NaCl (optimum 0.12 mol.L^−1^ NaCl). The genome is 1.67 Mbp with a G+C content of 55.5 mol%. Genome sequence annotation confirmed the absence of the methyl branch of the H_4_MPT Wood–Ljungdahl pathway, as described for other *Methanomassiliicoccales* members. Based on an average nucleotide identity analysis, we propose strain Mx-05^T^ as being a novel representative of the order *Methanomassiliicoccales*, within the novel family *Methanomethylophilaceae*, for which the name *Methanomethylophilus alvi* gen. nov, sp. nov. is proposed. The type strain is Mx-05^T^ (JCM 31474T).

## 1. Introduction

Only one species of the order *Methanomassiliicoccales* [1], *Methanomassiliicoccus luminyensis* B10^T^, has so far been isolated and described [2]. This archaeon was isolated from human feces and belongs to the family *Methanomassiliicoccaceae* which is predominantly found in soil and sediment environments according to 16S rRNA gene surveys [3,4]. *Methanomassiliicoccus luminyensis* is rarely detected in the gut environment [5,6] but was found to be one of the most widely distributed representatives of the *Methanomassiliicoccales* in a diverse range of environments, indicating a generalist lifestyle [7]. Accordingly, a second strain of *M. luminyensis* (strain CZDD1) was isolated from arsenic-contaminated paddy field soil [8]. A closely related species, ‘*Candidatus* Methanomassiliicoccus intestinalis’, was enriched from a human fecal sample [9] and is more prevalent in human populations than *M. luminyensis* [5,6]. Two additional enrichment cultures of *Methanomassiliicoccaceae* were generated from freshwater sediment [7] and peat soil [10]. A second family-level lineage within the *Methanomassiliicoccales* family was retrieved primarily from samples of the gut environment. Initially, it was variously referred to as “rumen cluster C” [11], “host-associated clade” [12], or “intestinal clade” [13]. In 2012, one of the first enrichment cultures of a representative of this lineage (strain Mx1201) was obtained and its genome was sequenced [14]. The name ‘*Candidatus* Methanomethylophilus alvus’ was proposed for this methanogenic archaeon [14,15] and the name ‘*Candidatus* Methanomethylophilaceae’ [16] was subsequently proposed for the family to which it belongs. Other enrichment cultures of *Methanomassiliicoccales* representatives belonging to ‘*Ca.* Methanomethylophilaceae’ were obtained from fecal and digestive tract samples of different animals including ruminants and termites [4,13,17,18,19], as well as from anaerobic digester sludge from garbage slurry [1]. 

*Methanomassiliicoccus luminyensis* B10^T^ and other *Methanomassiliicoccales* members characterized from enrichment cultures are hydrogenotrophic methyl-reducing methanogens (reducing methyl compounds into methane using H_2_ as the electron donor). Genomic analyses of *Methanomassiliicoccales* suggested different adaptations to the gut environment in ‘*Ca.* Methanomethylophilaceae’ members, such as the presence of specific adhesins and bile acid resistance genes [5,20]. *Methanomassiliicoccales* members display several unusual characteristics, including a split 16S and 23S rRNA gene operon, the absence of known histone-like proteins [21], and a new mode of energy conservation relying on a homologue of the respiratory complex I, which does not transfer electrons to a membrane-soluble electron carrier [19,22,23]. In addition, *Methanomassiliicoccus luminyensis* displays tetraether lipids with a butanetriol or a pentanetriol replacing one of the two glycerols [24,25]. *Methanomassiliicoccales* genomes encode a particular pyl system responsible for the biosynthesis of pyrrolysine (Pyl, the 22nd proteogenic amino acid) and its incorporation in methyltransferase enzymes required for methylamine-based methanogenesis [26,27].

The *Methanomassiliicoccales* members have attracted recent investigations because of their potential biotechnological applications, their roles in bioremediation and environmental element cycling, and their health relationships as part of the gut microbiome. For example, the tRNA^Pyl^–PylRS pair of ‘*Ca.* M. alvus’ has attracted growing interest in synthetic biology as it can incorporate several non-canonical amino acids in proteins with high specificity and efficiency [28]. The tRNA^Pyl^–PylRS pair of ‘*Ca.* M. alvus’ is already used to develop applications for human in vivo studies and cancer therapeutics [29]. The environmental relevance is exemplified by the important role played by *M. luminyensis* in the multi-species demethylation process of the toxic dimethylarsinic acid observed in paddy field soil [8]. The addition of *M. luminyensis* CZDD1 to an arsenic-contaminated paddy soil was shown to alleviate rice straighthead disease caused by dimethylarsinic acid [8]. *Methanomassiliicoccales* can also play an important role in animal and human gut microbiomes. Twenty-seven potential archaeal species have been identified in the human gut on the basis of average nucleotide identity (ANI) comparisons between 1167 genomes reconstructed from fecal metagenomes originating from five continents [6]. Of these, 12 belong to the *Methanomassiliicoccales*, 10 of which have the genetic potential to use trimethylamine as an electron acceptor for methanogenesis. Trimethylamine (TMA) is a bacterial metabolite produced from different dietary compounds (e.g., carnitine, lecithin) and is indirectly involved in the development of kidney [30] and cardiovascular [31] diseases, as well as trimethylaminuria [32]. It was, thus, proposed that *Methanomassiliicoccales* could play a beneficial role in human health by depleting this compound directly from its source [5,16]. Subjects with a higher abundance level of *Methanomassiliicoccales* in their gut have a lower TMA concentration in their feces than people without *Methanomassiliicoccales* [5]. Thus, we have proposed using these archaea [16] or genetically engineered bacteria expressing *Methanomassiliicoccales* genes [33] as probiotics. However, their therapeutic development and evaluation are impaired by the lack of isolated, well-characterized members of the host-associated clade of *Methanomassiliicoccales*. Beyond humans, a recent study on the distribution and abundance of gut methanogens within 250 animal species revealed that ‘*Ca.* Methanomethylophilaceae’ is one of the five main lineages of methanogens in this environment and can represent the large majority of the gut archaea in several animals [3]. 

Here, we report the isolation and formal taxonomic description of strain Mx-05^T^ as the second isolated member pertaining to the *Methanomassiliicoccales* order and propose the name *Methanomethylophilus alvi* gen. nov*.,* sp. nov., within the novel family *Methanomethylophilaceae* fam. nov. 

## 2. Materials and Methods

### 2.1. Medium of Enrichment, Isolation, and Maintenance

Yeast extract, tryptone and peptone were purchased from Becton Dickinson (Miami, FL, USA). All other chemicals were purchased from Sigma-Aldrich (Steinheim, Germany).

The enrichment culture was performed in a medium containing the following components per liter: KH_2_PO_4_ (0.5 g), MgSO_4_·7H_2_O (0.4 g), NaCl (0.4 g), NH_4_Cl (0.4 g), CaCl_2_·2H_2_O (0.05 g), sodium acetate (1 g), cysteine HCl (0.5 g), Na_2_S·9H_2_O (0.15 g), NaHCO_3_ (4 g), yeast extract (1 g), resazurin (1 mg), Widdel vitamin solution (20 mL) [34], Widdel trace element solution (1 mL) [34], and tungstate–selenite solution (1 mL) [2], methanol (60 mmol.L^−1^ final). The tungstate–selenite solution contained the following components per liter: NaOH (0.4 g), Na_2_SeO_3_ (2 mg), and Na_2_WO_4_·5H_2_O (4 mg). Before autoclaving, all chemicals except NaHCO_3_, Na_2_S·9H_2_O, and vitamins were added and the pH of the medium was adjusted to 7.5 using 10 mol.L^−1^ KOH. Subsequently, the medium was purged with oxygen-free N_2_ gas and transferred into Hungate tubes (5 mL per tube) and 100 mL serum bottles (30 mL per bottle). After autoclaving, methanol, NaHCO_3_, Na_2_S·9H_2_O, and vitamins were added to the medium. The tubes were pressurized by adding H_2_ after inoculation (200 kPa). 

The roll tube medium corresponded to the enrichment medium supplemented with 2% (*v*/*v*) of an *Eggerthella* sp. culture filtrate and 2% (*w*/*v*) agar. The roll tubes were pressurized with H_2_/CO_2_ (200 kPa) after inoculation. *Eggerthella lenta* was grown on commercial BHI medium (Sigma Aldrich), supplemented with sterile glucose (20 mM), Na_2_S 9H_2_O (0.4 g.L^−1^), NaHCO_3_ (2 g.L^−1^), and Widdel vitamin (20 mL.L^−1^) solutions after autoclaving. This culture was filtered and used when performing the serial dilution of the enrichment culture in solid medium-containing roll tubes.

The axenic culture (strain Mx-05^T^) was checked periodically by monitoring the cell morphology under a bright-field microscope and by confirming the absence of fermentative contaminants when inoculating the modified BHI medium in the absence of H_2_ and methanol.

Strain Mx-05^T^ was then cultured in a medium containing the following per liter: KH_2_PO_4_ (0.5 g), MgSO_4_·7H_2_O (0.4 g), NaCl (5 g), NH_4_Cl (0.4 g), CaCl_2_·2H_2_O (0.05 g), sodium acetate (1 g), yeast extract (2 g), tryptone (2 g), peptone (2 g), casamino acid (2 g), cysteine HCl (0.5 g), Na_2_S·9H_2_O (0.15 g), NaHCO_3_ (4 g), yeast extract (1 g), resazurin (1 mg), Widdel vitamin solution (20 mL) [34], Widdel trace element solution (1 mL) [34], tungstate-selenite solution (1 mL), methanol (60 mmol.L^−1^ final), and a filtrate of *Eggerthella lenta* DSM 2243^T^-grown culture (10% *v*/*v*).

### 2.2. Genome Sequencing, Genomic and Phylogenomic Analyses

The genome of strain Mx-05^T^ was sequenced on an Illumina Hiseq instrument generating 2 × 150 bp reads. The reads were assembled with the GS De Novo Assembler v11/2015 (Roche) into a single circular chromosome. The genome was annotated with the Prokaryotic Genome Annotation Pipeline [35]. We built a phylogenomic tree of the *Methanomassiliicoccales* family using 41 phylogenetic marker proteins [36] from 54 *Methanomassiliicoccales* representatives, including Mx-05^T^, ‘*Ca.* M. alvus’ Mx1201, *M. luminyensis* B10^T^, and eight genomes derived from previously described enrichment cultures, as well as from 43 genomes reconstructed from metagenomes (MAGs), covering the currently known phylogenetic diversity of the order (at least one genome per candidate genus in GTDB [37]). The tree was rooted with three members of ‘*Ca.* Sysuiplasmatales’, the closest relatives to *Methanomassiliicoccales* family that do not carry *mcrABG* genes [38]. The protein sequences used to build the tree were retrieved with an HMM search (hmmer v3.3.2 [39]), aligned with MAFFT (v7.453) [40], trimmed with BMGE (v2) [41], and concatenated. A maximum likelihood tree was built with IQ-TREE (v2.0.6) [42] using the LG+F+R10 model. For the 16S rRNA gene tree, we extracted the 16S rRNA gene from the Mx-05^T^ genome and other genomes selected for the phylogenomic analysis using Metaxa2 (v2.2) [43]. The maximum likelihood tree was built with IQ-TREE using the GTR+F+R4 model.

### 2.3. Microscopy 

The cells were observed with an inverted contrasting microscope (Leica DMIRB, Leica, Germany) equipped with a Hamamatsu C13440 camera. For the cryo-electron microscopy (Cryo-EM), 4 μL of each sample were deposited on Quantifoil R2/1 glow discharge, then cryofixed using a Leica EMGP (Leica, Germany). The samples were visualized under a Tecnai F20 electron microscope (ThermoFisher, Waltham, MA, USA) with low-dose conditions and imaged using a Falcon II camera (ThermoFisher, Waltham, MA, USA). 

### 2.4. Physiological Characterization

Test substrates for methanogenesis were added to the culture medium after autoclaving. In addition to H_2_/methanol (60 mmol.L^−1^), the tested substrates were trimethylamine (20 mmol.L^−1^), dimethylamine (20 mmol.L^−1^), monomethylamine (20 mmol.L^−1^), dimethyl sulfide (20 mM), CO_2_ (20% of the gas phase), formate (20 mmol.L^−1^), ethanol (20 mmol.L^−1^), and acetate (20 mmol.L^−1^). Trimethylamine, dimethylamine, monomethylamine, dimethyl sulfide, and CO_2_ were tested as potential electron acceptors in the presence of H_2_. Formate and ethanol were tested as electron donors for methanol reduction in the absence of H_2_. Acetate was tested solely as an energy source in the absence of H_2_.

The temperature range and optimum value for Mx-05^T^ were determined at pH 7 and with 0.09 mol.L^−1^ of NaCl, from 25 °C to 50 °C, with a 5 °C increment and measured at 37 °C. The pH range and optimum value were determined at 37 °C and with 0.09 mol.L^−1^ of NaCl, from pH 5.3 to pH 9.5, with 0.2–0.3 pH steps. The salinity range and optimum value were determined at 37 °C and pH 7, from 0 to 0.86 mol.L^−1^ NaCl. 

The susceptibility to lysis with sodium dodecyl sulphate (SDS) was tested by adding SDS at a final concentration of 0.01% to a late-logarithmic-phase culture of Mx-05^T^ and by following the decrease in OD. The susceptibility to lysis due to osmotic stress was also tested by resuspending cells in distilled water.

## 3. Results and Discussion

### 3.1. Selective Enrichment and Isolation

**Figure 1:**
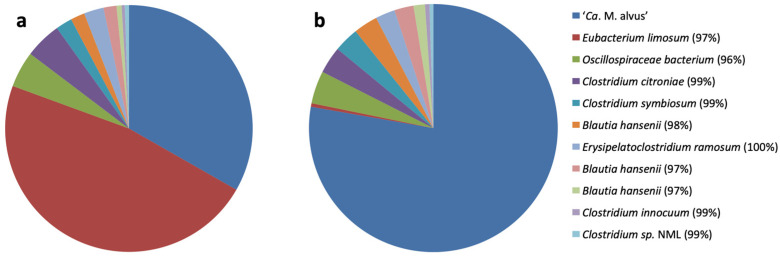
Proportional abundance of bacteria and archaea in enrichment cultures using either H_2_/methanol (**a**) or H_2_/trimethylamine (**b**) two years after the beginning of the enrichment culture. Percentage values next to the bacterial names correspond to the sequence identity they display to the closest species.

The inoculum for the enrichment culture was a fecal sample from an elderly subject [14]. The absolute abundances of total bacteria and *Methanomassiliicoccales* from “host-associated” and “free-living” clades were followed by qPCR three days after each transfer, using primers and conditions previously described [5]. Transferring the culture every three days led to the enrichment of ‘*Ca.* M. alvus’ [14], while transfers every 10 days led to the enrichment of ‘*Ca.* M. intestinalis’ [9] from the same initial sample. The latter was enriched up to 45% of the whole microbial community but it was lost after two years of subculturing. The 16S rRNA gene amplicon sequencing (MiSeq) was performed on enrichment cultures of the “host-associated” clade, amended with H_2_ as the electron donor and either methanol or trimethylamine as terminal electron acceptors, following the procedure described by Flemer et al. [44]. The enrichment culture on H_2_/trimethylamine had the highest relative abundance of *Methanomassiliicoccales* (Figure 1). 

The main difference in the compositions of the two enrichment cultures was due to the massive out-growth of a species closely related to *Eubacterium limosum* in the presence of methanol, with all other bacteria being present in similar relative abundance levels between the two conditions. *Eubacterium limosum* can use methanol and possibly H_2_ as sources of energy, making it a major competitor of the *Methanomassiliicoccales* members in the culture conditions that we used and potentially in the gut microbiome. After additional transfers (every three days for two years) and filtrations through 0.45 µm to deplete *Eubacterium* sp., ‘*Ca.* M. alvus’ Mx1201 represented 99% of the cells in the enrichment culture while retaining the presence of one rod-shaped microorganism. The first attempts to obtain the methanogen in axenic culture on a solid medium failed, which we hypothesized might be due to its dependence on bacteria for one or more growth factors. The remaining microbial contaminant was further isolated and was identified as an *Eggerthella* strain sharing more than 97% identity similarity with *Eggerthella lenta*. Thereafter, a filtrate (0.2 µm) from a culture supernatant of *Eggerthella sp.* was added to the roll tube solid medium during the process of the isolation of the methanogen. After one month of incubation at 37 °C, white and round colonies measuring 0.5–1 mm in diameter were obtained, transferred to a fresh liquid medium, and serially diluted. The addition of the culture filtrate of *Eggerthella* sp. was, therefore, essential to obtain the methanogenic strain Mx-05^T^ in axenic culture.

### 3.2. Phylogeny and Genome Features

**Figure 2:**
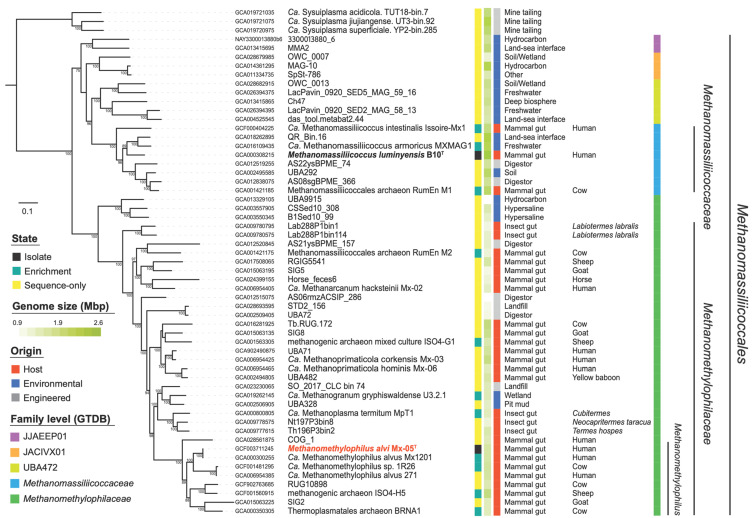
*Methanomassiliicoccales* maximum likelihood tree built with an LG+F+R10 model from 41 concatenated protein sequences. The tree is rooted on ‘*Candidatus* Sysuiplasmatales’ [38], with 10,325 positions after trimming. Values on the tree indicate the bootstrap supports > 70%. The tree was formatted with Itol [45]. Isolated species are indicated in bold.

Mx-05^T^ is a member of a clade that is composed almost exclusively of organisms from the gut microbiome [5,12] corresponding to taxa of the previously proposed ‘*Ca.* Methanomethylophilaceae’ (Figure 2). As expected, Mx-05^T^ and ‘*Ca.* M. alvus’ Mx1201 are contiguous in the tree. Mx-05^T^ is closely related to an MAG obtained from a human gut metagenome (GCA_006954385) [5] and another taxon obtained from a rumen enrichment culture, ‘*Ca.* Methanomethylophilus sp.’ 1R26 (GCF_001481295) [18]. Similar placements of Mx-05^T^ and similar tree topologies for the *Methanomassiliicoccaceae* and proposed *Methanomethylophilaceae* families were obtained within a 16S rRNA gene tree (Figure S1). Mx-05^T^ and *M. luminyensis* B10^T^ share 86.9% nucleotide identity similarity on the 16S rRNA and 76.2% amino acid identity similarity on the McrA sequence. They share 66.3% ANI similarity with an alignment of 12% on the whole genome of Mx-05^T^.

The Mx-05^T^ genome is 1.67 Mbp in size with a G+C content of 55.5 mol%. Both the genome size and G+C content are smaller than those of *M. luminyensis* B10^T^ (Table 1). More generally, the genome length within the proposed *Methanomethylophilaceae* family (average of 1.57 Mbp) is substantially smaller than that of the *Methanomassiliicoccaceae* (average of 2.12 Mpb, Mann–Whitney *p* = 0.0001, Figure 2), suggesting reductive genome evolution during the adaptation to the gut environment. The Mx-05^T^ genome codes for 1597 predicted CDS, 45 tRNA genes, one 23S gene, one 16S gene, and two 5S rRNA genes. The rRNA genes do not form a single operon in Mx-05^T^, which is a characteristic shared with other members of the ‘*Ca.* Thermoplasmatota’ phylum [21,46]. Mx-05^T^ genome has only 27 single-nucleotide polymorphisms (SNPs), with the genome of ‘*Ca.* M. alvus’ Mx-1201 obtained from the initial enrichment in 2012 (CP004049.1/GCA_000300255.2) and a 32 bp insertion in the 5’ end of a hypothetical pyrrolysine-containing protein [21]. Twenty of the SNPs correspond to synonymous mutations, three are in non-coding regions, one is in the CRISPR repeat array, and one led to the deletion of the 5’ end of a gene coding for a GT2/GT4 glycosyltransferase. Accordingly, genomic features previously described for ‘*Ca.* M. alvus’ Mx1201 are shared by Mx-05^T^ [12,27,47]. These include the lack of genes coding for the methyl branch of the H_4_MPT Wood–Ljungdahl pathway, which is involved in (i) the reduction of CO_2_ into a methyl group during hydrogenotrophic CO_2_-reducing methanogenesis (H_2_ + CO_2_ → CH_4_ + H_2_O) and (ii) the oxidation of a methyl group into CO_2_ during methylotrophic methanogenesis (e.g., 4 CH_3_OH → 3CH_3_ + CO_2_). Mx-05^T^ codes for McrABG involved in methane production, for several methyltransferases used to transfer methyl groups from various substrates to coenzyme M (i.e., methanol (MtaBC), monomethylamine (MtmBC), dimethylamine (MtbBC), trimethylamine (MttBC), and methylated thiol compounds (MtsAB)), as well as [NiFe]-hydrogenase (MvhAG) to use H_2_ as an electron donor. The Mx-05^T^ genome harbors genes for six predicted membrane-bound adhesin-like proteins with multiple Flg_New repeats, including two (AYQ55217.1 and AYQ55598.1) that are longer than 1000 amino acids. Flg_New repeats have been identified in the Pil3 protein of *Streptococcus gallolyticus*, playing an essential role in its adhesion to the human gut mucosa [48]. Proteins with Flg_New repeats were also identified in distantly related gut *Methanimicrococcus* (*Methanosarcinales*) members [47], as well as in viruses of gut *Methanomassiliicoccales* members [49]. These proteins likely play an important role in the gut colonization of Mx-05^T^. Other characteristics are the absence of characterized histone or other nucleotide-associated proteins (NAPs) involved in the formation of chromatin. A large diversity of genes coding for these NAPs have been reported in other members of the ‘*Ca.* Thermoplasmatota’ phylum, including Alba, MC1, and HU [50]. This highlights a potential loss of the histone gene in the last common ancestor of the ‘*Ca.* Thermoplasmatota’ phylum, which was then replaced by various enzymes. *M. luminyensis* codes for an Alba protein but it is not expressed at a detectable level [50]. A potential new NAP was identified in ‘*Ca.* M. alvus’ Mx1021 [50] and corresponded to AYQ55638.1 in Mx-05^T^. 

### 3.3. Morphology

**Figure 3:**
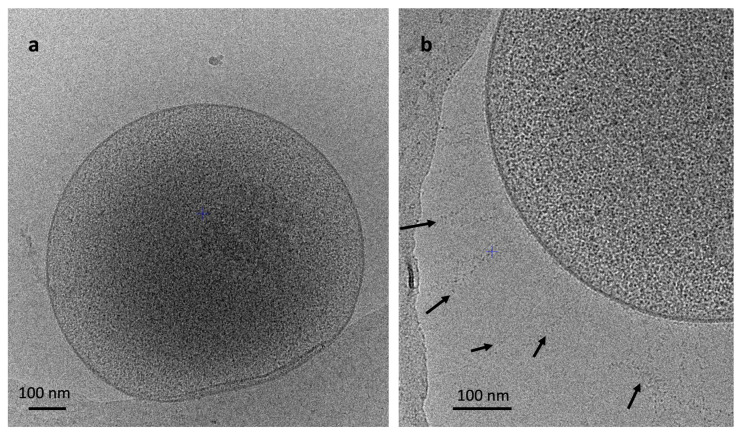
Cryo-electron microscopy pictures of strain Mx-05^T^ showing the whole cell (**a**) and details (**b**). Black arrows (**b**) indicate potential adhesin-like proteins on the surface of the cell.

Mx-05^T^ cells are cocci measuring 0.4–0.7 μm that are not autofluorescent when excited at the 420 nm and 350 nm wavelengths. This absence of autofluorescence matched the absence of the genes coding for the F_420_ biosynthesis pathway in the Mx-05^T^ genome. Under Cryo-EM microscopy (Figure 3), we did not observe a flagellum (archaeallum), concordant with the absence of the corresponding genes in the genome. Long fibrillar appendices (up to 300 nm) are present on the cell surface (Figure 3b), which we speculate to represent the long adhesin-like proteins with Flg-new/List-Bac repeat domains (PF09479) coded by the genome of Mx-05^T^. No cell wall or S-layer structure was observed, which agrees with the results of cell lysing in SDS (0.01% (*w*/*v*)) and in distilled water. In contrast to *M. luminyensis* B10^T^ cells, which were reported to have two membranes [2], we only observed a single membrane in Mx-05^T^ cells. 

### 3.4. Physiology

Mx-05^T^ is a strict anaerobe that uses methyl compounds as electron acceptors while oxidizing H_2_ for methanogenesis. The methyl compounds used for methanogenesis comprise methanol, monomethylamine, dimethylamine, and trimethylamine but not dimethyl sulfide. The Mx-05^T^ genome has homologues of *mtsAB* genes that are involved in the utilization of several methylated thiol compounds for methanogenesis in *Methanosarcina barkeri* [51]. Those genes are likely not involved in the transfer of methyl from dimethylsulfide to coenzyme M in Mx-05^T^, as neither growth nor methane production was observed when dimethylsulfide and H_2_ were added to the culture medium. Instead, MtsAB homologues may catalyze methyl transfer from other types of methylated thiol to be tested (e.g., methylthiol, dimethylsulfoniopropionate). No growth was observed in the absence of H_2_. Formate and ethanol were not used as electron donors to reduce methyl compounds. Formate, H_2_/CO_2_, and acetate were not used for methanogenesis. 

The growth of Mx-05^T^ required an unknown factor that could be supplied by the culture supernatant filtrate of an *Eggerthella* sp., which had been isolated from the initial enrichment culture. A similar growth enablement was obtained using *E. lenta* DSM 2243^T^. The growth rate of Mx-05^T^ was correlated with the proportional volume (2–10% (*v*/*v*)) of *E. lenta* supernatant filtrate added to the culture. The unknown growth factor could also be provided by rumen fluid, thereby indicating that *E. lenta* and possibly other bacteria growing in the rumen produce the factor(s) required by Mx-05^T^. In comparison to the *E. lenta* culture filtrate, the growth of Mx-05^T^ was not influenced by the volume of rumen fluid added to the medium (in the range of 2–10% (*v*/*v*)), thereby indicating that the unknown growth factor is present at a higher concentration in the rumen fluid than in the *E. lenta* filtrate. The unknown factor does not appear to be sensitive to temperature, as the growth of the methanogenic isolate was similar when using an autoclaved culture filtrate of *E. lenta*.

The growth of Mx-05^T^ was observed between 30 °C and 40 °C, with an optimum temperature at 37 °C. The pH range for growth was between 6.9 and pH 8.3, with an optimum value found at pH 7.5. At pH 6.9, Mx-05^T^ grows almost as well as at pH 7.5, although a slightly lower pH is strongly inhibitory. Regarding salinity, Mx-05^T^ can grow in conditions of between 0.02 and 0.34 mol.L^−1^ NaCl, with an optimum value of around 0.12 mol.L^−1^ NaCl. Mx-05^T^ grows in conditions almost similar to those of *M. luminyensis* (Table 1) [2]. Under optimal conditions (37 °C, pH 7.5, and 0.09 mmol.L^−1^ NaCl), the maximum specific growth rate of Mx-05 ^T^ was 0.026 h^−1^ (i.e., doubling time of 26.7 h).

## 4. Conclusions

Metagenomic and metataxonomic studies have revealed the global environmental distribution of *Methanomassiliicoccales* representatives [4,7,52,53] and a particular association with the gut microbiomes of animals [3,4,19], including ruminants [54,55] and humans [5,6,56]. Over the last 10 years, multiple enrichment cultures of *Methanomassiliicoccales* have been described [1,4,7,9,10,13,14,17,18], although only one generalist member (*M. luminyensis* B10^T^) of this order has been obtained in axenic culture and described so far [2]. Here, we report the description of a second hydrogenotrophic methyl-reducing methanogen representative of this order (strain Mx-05^T^), being the first isolate of a family-level clade associated with the gut microbiome of various animals. Based on physiological, phylogenetic, and genetic features, we propose strain Mx-05^T^ as pertaining to a novel archaeal genus designated as *Methanomethylophilus* gen. nov., and named as *M. alvi* sp. nov. within the family *Methanomethylophilaceae* fam. nov. The originally proposed Candidatus name for this species (*M. alvus*) was changed to *M. alvi* following a recommendation by Oren et al. [15]. In contrast to *M. luminyensis* B10^T^, *Methanomethylophilus alvi* Mx-05^T^ requires one or multiple unknown growth factors elaborated by *Eggerthella lenta*, of the family *Eggerthellaceae*, whose members are also recognized as members of the mammalian gut microbiome [57,58]. This requirement highlights the inter-species dependencies that can occur between members of the gut microbiota and warrants further biochemical and molecular investigations. The addition of the culture supernatant filtrate from the last co-cultured bacterium in the enrichment was critical to the isolation of *M. alvi* Mx-05^T^, as it eliminated the need to have this live bacterium in culture. Such a strategy could enable the isolation of other *Methanomassiliicoccales* members from animal or human gut microbiomes, which have so far only been obtained in mixed enrichment cultures, or more largely for other microorganisms that remain recalcitrant to isolation. Interestingly, in the case of *M. alvi* Mx-05^T^, these unknown growth factors were also present in rumen fluid, where they most probably result from microbial activities and may explain the presence of *Methanomethylophilus* spp. in both the ruminant and human gut environments. Further studies on *M. alvi* Mx-05^T^ could be relevant for different human health aspects, such as its utilization as a probiotic (archaebiotics, [16]) either to prevent the harmful effects of trimethylamine on human cardiovascular health or to develop methane mitigation strategies in ruminants.

### 4.1. Description of Methanomethylophilus gen. nov.

*Methanomethylophilus* (Me.tha.no.me.thy.lo’phi.lus. N.L. pref. *methano-*, pertaining to methane (in compound words); N.L. neut. n. *methyl*, pertaining to the methyl group; N.L. masc. adj. suff. *-philus*, loving, from Gr. masc. adj. *philos*, on; N.L. masc. n. *Methanomethylophilus*, methane-producing organism-loving methyl groups). Strictly anaerobic and methanogenic cells. They only obtain energy by reducing methyl compounds into methane using H_2_ as the electron donor (methyl-reducing hydrogenotrophic cells). Predominantly living in the intestinal microbiome. The type species is *Methanomethylophilus alvi*.

### 4.2. Description of Methanomethylophilus alvi sp. nov.

*Methanomethylophilus alvi* (al′vi. L. gen. n. *alvi* of the bowels). 

In addition to the characters described for the genus, the species is characterized by the following properties. The cells are single-membrane cocci measuring 0.4–0.7 µm, occurring alone. The cells lyse rapidly in SDS (0.01%) and in distilled water. Motility is not observed and genes coding for the archaellum are absent. Growth occurs between 30 °C and 40 °C (optimum, 37 °C), between pH 6.9 and 8.3 (optimum pH 7.5), and between 0.02 and 0.34 mol.L^−1^ NaCl (optimum 0.12 mol.L^−1^ NaCl). Obligate anaerobe. Produces methane using hydrogen as an energy source and methanol, trimethylamine, dimethylamine, or monomethylamine only as electron acceptors. Requires the addition of the filtrate of a grown culture of *Eggerthella* sp., *Eggerthella lenta,* or rumen fluid for growth.

The type strain Mx-05^T^ (=JCM 31474^T^) was isolated from a fecal sample of an elderly subject.

### 4.3. Description of Methanomethylophilaceae fam. nov.

*Methanomethylophilaceae* (Me.tha.no.me.thy.lo.phi.la’ce.ae. N.L. fem. pl. n. *Methanomethylophilus* is the type genus of the family; -aceae ending to denote a family; N.L. fem. pl. n. *Methanomethylophilaceae* the family of the genus *Methanomethylophilus*).

Mesophilic methanogens non-motile. Found mostly in the animal digestive tract. Obligate anaerobes. All sequenced members lack the genes of the H_4_MPT-methyl branch of the Wood–Ljungdahl pathway and likely cannot disproportionate methyl compounds into methane and CO_2_ or reduce CO_2_ while oxidizing H_2_. Most have genes coding for methyltransferases involved in the transfer of methyl groups from methyl compound substrates to coenzyme M and genes coding for hydrogenases to reduce the methyl groups of these compounds using H_2_ as electron donor.

The type genus is *Methanomethylophilus*.

This article is dedicated to the American microbiologist Professor R.A. Mah, for his important contribution to the physiology and taxonomy of methanogens.

## 5. Patents

One patent resulted from the work reported here, under the reference WO2019106181A1.

## Figures and Tables

**Table 1:** Comparison of characteristics of strains Mx-05^T^ (**1**) and *Methanomassiliicoccus luminyensis* B10^T^ (**2**).

Characteristics	1	2
Source	Human feces	Human feces
Genome length (bp)	1,666,767	2,637,810
DNA G+C content (mol%)	55.5	59.93
Morphology	Cocci, as single cells	Cocci, as single cells
Diameter (µm)	0.4–0.7	0.7–1.0
Motility	Non-motile	Non-motile
Growth temperature		
Range	30–40	30–40
Optimum	37	37
Growth pH		
Range	6.9–8.3	7.2–8.4
Optimum	7.5	7.6
Growth NaCl (mol.L^−1^)		
Range	0.02–0.34	0.02–0.26
Optimum	0.12	0.17
Required growth factor	Filtrate from a grown *E. lenta* or rumen fluid	acetate
Substrates for methane production	H_2_/methanol H_2_/monomethylamine H_2_/dimethylamine H_2_/trimethylamine	H_2_/methanol H_2_/monomethylamine H_2_/dimethylamine H_2_/trimethylamine
Lysed by osmotic shock/SDS	yes	yes

## Data Availability

The GenBank accession number for the complete genome sequence of *Methanomethylophilus alvi* Mx-05^T^ is CP017686.1. The strain was deposited at the Japan Collection of Microorganisms (JCM) under the number JCM 31474T.

## Supplementary Materials

The following supporting information can be downloaded at: https://www.mdpi.com/article/10.3390/microorganisms11112794/s1. Figure S1: Position of Mx-05^T^ in the 16S rRNA gene phylogeny of the *Methanomassiliicoccales*. Maximum likelihood tree built with GTR+F+R4 model. The tree is rooted on ‘*Candidatus* Sysuiplasmatales’ [38], with 1550 positions after trimming. Values on the tree indicate bootstrap support > 70%.
