# Supplementary material for: Methanomethylophilus alvi gen. nov., sp. nov., a Novel Hydrogenotrophic Methyl-Reducing Methanogenic Archaea of the Order Methanomassiliicoccales Isolated from the Human Gut and Proposal of the Novel Family Methanomethylophilaceae fam. nov
Source: Microorganisms. 2023 Nov 17;11(11):2794. doi: 10.3390/microorganisms11112794 (PMC10673518; doi:10.3390/microorganisms11112794)
Supplement: Supplementary file 1 [file microorganisms-11-02794-s001.zip › microorganisms-2714981-supplementary.pdf]

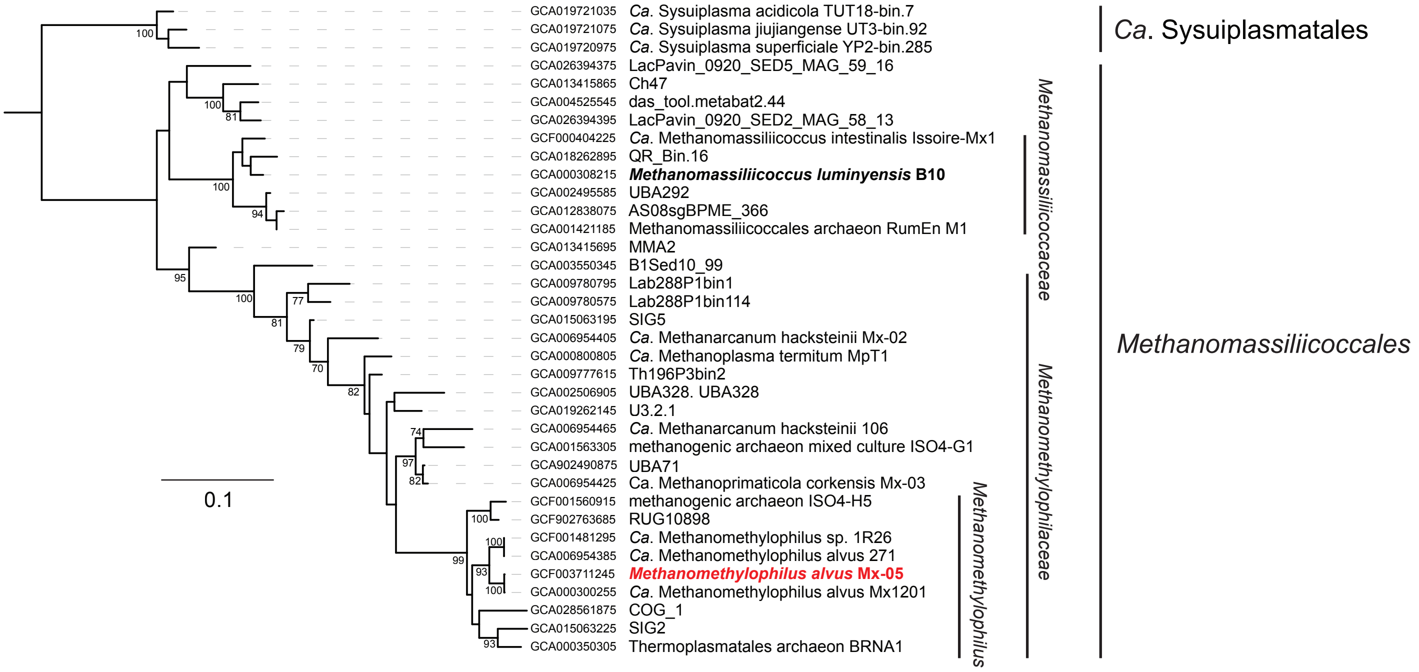

Figure S1: Position of Mx-05T in the 16S rRNA gene phylogeny of the Methanomassiliicoccales. Maximum likelihood tree built with GTR+F+R4 model. The tree is rooted on 'Candidatus Sysuiplasmatales' [38], with 1550 positions after trimming. Values on the tree indicate bootstrap support > 70%.
